# A multidimensional nomogram combining clinical factors and imaging features to predict 1-year recurrence of low back pain with or without radicular pain after spinal manipulation/mobilization

**DOI:** 10.1186/s12998-023-00500-5

**Published:** 2023-08-10

**Authors:** Dai Sun, Yang-yang Liu, Dan Luo, Ye-qi Wu, Zhi-qiang Yan, Yun-qi Liang, Xue-yan Huang, Jia-long Lin, Hua-song Luo, Rui Wang

**Affiliations:** 1 Department of Massage, Hangzhou TCM Hospital Affiliated to Zhejiang Chinese Medical University, Hangzhou, China; 2 The First Affiliated Hospital of Zhejiang Chinese Medical University, Hangzhou, China

**Keywords:** Low back pain, Spinal manipulation/mobilization, Recurrence, Nomogram, Prognosis

## Abstract

**Background:**

In this retrospective study, we aimed to develop a nomogram to predict recurrence during a 1-year period of spinal manipulation/mobilization (SM/M) in patients with low back pain (LBP) with greater pain intensity, more severe comorbid conditions, or a neuropathic component.

**Methods:**

A total of 786 consecutive patients with LBP treated with SM/M as primary therapy were divided into training (n = 545) and validation (n = 241) sets. Cox regression analyses were used to assess the relative value of clinical factors and lumbar magnetic resonance imaging features associated with recurrence during the 1-year period. Predictors of recurrence with significant differences were used to construct a nomogram in the training set. We evaluated the performance of the model on the training and validation sets to determine its discriminative ability, calibration, and clinical utility. The prognostic value of the nomogram for predicting recurrence was assessed using Kaplan–Meier analysis and time-dependent receiver operating characteristic analyses.

**Results:**

A nomogram comprising hospitalization time, previous history of LBP, disease duration, lumbar range of motion, lower extremity tendon reflex, muscle strength, ratio of herniation to uncompressed dural sac area, and Pfirrmann classification was established for recurrence during a 1-year period after SM/M in patients with LBP. Favorable calibration and discrimination were observed in the nomogram training and validation sets (C-index 0.753 and 0.779, respectively). Decision curve analysis confirmed the clinical utility of the nomogram. Over a 1-year period, the nomogram showed satisfactory performance in predicting recurrence in LBP after SM/M.

**Conclusion:**

We established and validated a novel nomogram that can accurately predict a patient's risk of LBP recurrence following SM/M. This realistic prognostic model may aid doctors and therapists in their decision-making process and strategy optimization for non-surgical treatment of LBP using SM/M.

**Supplementary Information:**

The online version contains supplementary material available at 10.1186/s12998-023-00500-5.

## Background

Low back pain (LBP) is a prevalent cause of disability worldwide [1]. At the pan-European level approximately 40% of the population experiences pain within a 12-month period [2]. LBP is also one of the top 20 public health issues in China [3]. The global burden of LBP—in terms of incidence, healthcare expenditure, and indirect costs related to lost workdays or reduced productivity—is substantial. Many people experience benign or mild LBP, which is often self-limiting. However, for a few people with greater pain intensity, more severe comorbid conditions, or a neuropathic component it is associated with a poorer prognosis [4]. Lumbar magnetic resonance imaging (MRI) is recommended for these patients to verify the presence of herniated discs or other degenerative changes as the cause of pain [5]. The treatment and prevention of LBP recurrence has become a clinical challenge.

Recently, with greater concordance among international guidelines on LBP, non-invasive treatment has become the dominant option, including passive treatment, drug-based therapy, physiotherapy, and exercise [6]. However, LBP management remains heterogeneous among countries. This is because LBP patients typically present with multifactorial pathologies and comorbidities that often require a multimodal analgesic approach [7, 8]. Spinal manipulation/mobilization (SM/M) is one of the most popular techniques used by physiotherapists and is often used as an adjunct to conventional LBP treatments [9, 10]. In China, specifically, SM/M is commonly used as part of traditional Chinese medicine, and so Chinese clinicians expected its clinical effectiveness in modern practice as well [11, 12].

SM/M works by improving the mobility of the spine and hips to reduce pain and dysfunction, and the core operations include both mobilization and manipulation [9]. Mobilization uses a low-grade velocity passive movement technique within the patient's controllable range of motion to achieve spinal stretching, while manipulation uses a high-velocity, short-amplitude impulse or thrust applied to the synovial joint at or near the limits of physiological motion [9]. The hypothesis of how SM/M works can be roughly divided into biomechanical and neurophysiological hypotheses. The modes of action may be to reduce mechanical stress within the spine [13] or to affect major afferent neurons and motor control systems from paraspinal tissues [14]. Many recent guidelines that recommend SM/M emphasize the importance of targeting the appropriate individuals for treatment, particularly for patients with the more severe symptoms described above [15–17]. Unfortunately, there are no predictive models to determine which patients with LBP should be advised to use SM/M.

Hence, in this study, we aimed to develop and validate a novel multidimensional nomogram which could predict 1-year recurrence of LBP after SM/M.

## Methods

The following article was prepared in accordance with the Transparent Reporting of a Multivariable Prediction Model for Individual Prognosis or Diagnosis (TRIPOD) reporting checklist (Additional file 1: Appendix S1).

### Study design

The study protocol was approved by the Ethics and Human Participants Committee (No.2022KY052), and the requirement for informed consent was waived because of the retrospective nature of this study. We screened the clinical records of consecutive inpatients with LBP with or without radicular pain who were treated at the Hangzhou TCM Hospital, affiliated with the Zhejiang Chinese Medical University, from November 2014 to October 2021. Patients who were recommended non-surgical treatment since their neurological examination did not reveal sphincter incontinence or foot drop during were identified.

Patients were included if they met the following criteria: (1) aged between 18 and 70 years; (2) no restrictions on sex or occupation; (3) LBP with or without radicular pain [18–20], and confirmed MRIs showed varying degrees of lumbar disc herniation or degeneration; (4) SM/M was the main treatment option but could be combined with a variety of conservative management protocols (Additional file 2: Appendix S2A-2B); (5) over 1 week in the hospital with sufficient information in their records; and (6) complete and clear MRIs for measurement. The exclusion criteria were as follows: (1) MRIs suggestive of spinal stenosis due to lumbar spondylolisthesis or ligamentum flavum hypertrophy; (2) lumbar surgery, trauma, tumor, spinal infection, or systemic rheumatological disease; (3) SM/M less than 3 times; and (4) incomplete follow-up.

### Patient characteristics and MRI variables

Demographic data including age, sex, occupation, body mass index (BMI), occupation and chronic health problems such as diabetes, hypertension, and cardiopathy were reviewed using medical records. Additionally, information was collected on overall pain scores using the numerical rating scale (NRS), radicular pain in the lower extremities, tendon reflexes and muscle strength, lumbar range of motion, straight leg raise test on admission, and whether a combined epidural was used.

All patients were scanned with a 1.5 T MRI scanner (Ingenia, Philips Healthcare, Best, Netherlands), and sections were obtained at a thickness of 4 mm in both the axial and sagittal planes. Routine intervertebral disc protocols consisted of sagittal T1-weighted (T1WI) and T2-weighted (T2WI) images. T2WI images were obtained by axial MRI scanning with the vertebral body aligned parallel to the inferior endplate. The following qualitative imaging parameters were independently assessed by both a clinician and a radiologist [21]: (1) characteristics of the disc herniation; (2) apical location of herniation; (3) nerve root impingement; (4) the ratio of intraspinal herniation area (Additional file 3: Appendix S3A-3B); (5) the ratio of herniation to uncompressed dural sac area (Additional file 3: Appendix S3C-3D); (6) Pfirrmann classification determined by assessing the T2WI signal intensity of the epidural material and maximum height of the intervertebral disc in the sagittal plane (Additional file 3: Appendix S3E-3F) [22]. Assessment of the axial plane was limited to the segment of the largest disc herniation. All measurements were performed using the Picture Archiving and Communication System. Images were read by two clinical experts in spinal MRI interpretation, one was a musculoskeletal radiologist with subspecialty experience in spinal imaging, and the other was a clinician. Differences were resolved through either discussion or by a third researcher.

### Statistical analysis and construction of the nomogram

All statistical analyses were performed using SPSS for Windows (version 17.0; Chicago, IL, USA) and R software (version 4.0.1; https://www.r-project.org/). All eligible samples were randomly separated into the training and validation (7:3) groups using the R caret package. For continuous data with a normal or an abnormal distribution, the Student’s t- and Wilcoxon Mann–Whitney U test were used to analyze the statistical significance of differences between the with and without recurrence groups. Categorical variables were compared using the chi-squared or Fisher’s exact test. The reported statistical significance levels were two-sided, and statistical significance was set at P-values less than 0.05.

In the training set, univariate analyses based on clinical characteristics and imaging features were performed using SPSS software. Variables that achieved significance (*P* < 0.05), and those that were non-significant but clinically important, were entered into the multivariable analyses via the Cox regression model. Based on the results of the multivariable analysis, a nomogram was formulated using the survival and rms packages of R software. Backward stepwise selection was performed using the likelihood ratio test with Akaike’s information criterion as the stopping rule [23].

### Validation, calibration, and clinical utility of the nomogram

Predicted values were calculated for each individual in the validation set according to the formula constructed using the training set. The predictive discrimination of the nomogram was assessed using the ROC curve and area under the curve (AUC) [24]. The performance of the logistic regression model for predicting outcomes was assessed by calculating the concordance index (C-index) based on an AUC of 1.0, which indicates that the nomogram provides full discrimination [24]. Model C-indices for the different subgroups were based on previously described methods. The agreement between the observed and predicted values was assessed using calibration curves in both the training and validation sets, which would ideally perfectly align with the diagonal reference line [25].

To evaluate the clinical utility of the nomogram, decision curve analysis (DCA) was performed to calculate the net benefits at different threshold probabilities in the full dataset, combining the training and validation sets [26]. We sought to demonstrate the independent predictive ability of the nomogram beyond LBP recurrence after SM/M. To validate risk stratification using our established nomogram in terms of recurrence-free probability scores, we calculated each patient using our nomogram model [0.31583*(hospitalization time. continuous < 14 days) + 0.12068*(previous history of LBP = positive) + 0.13391*(disease duration. continuous < 0.45 months) + 0.05705*(lumbar range of motion = restricted) + 1.08123*(lower extremity tendon reflex = weakness) + 0.39387*(lower extremity muscle strength = weakness) + 0.59307*(ratio of herniation to uncompressed dural sac area. continuous < 0.0458%) + 0.26210*(Pfirrmann classification = Grade IV and V) + 0.62325*(Pfirrmann classification = Grade VI and VII)] and determined cutoff values by receiver operating characteristic (ROC) analysis as the optimal threshold, and Kaplan–Meier survival analysis was performed [24]. Time-dependent ROC curves were plotted to evaluate the performance of the predictive nomogram for 3-, 6-, and 9-month recurrence after SM/M [27, 28].

## Results

### Study flowchart and population characteristics

**Fig. 1:**
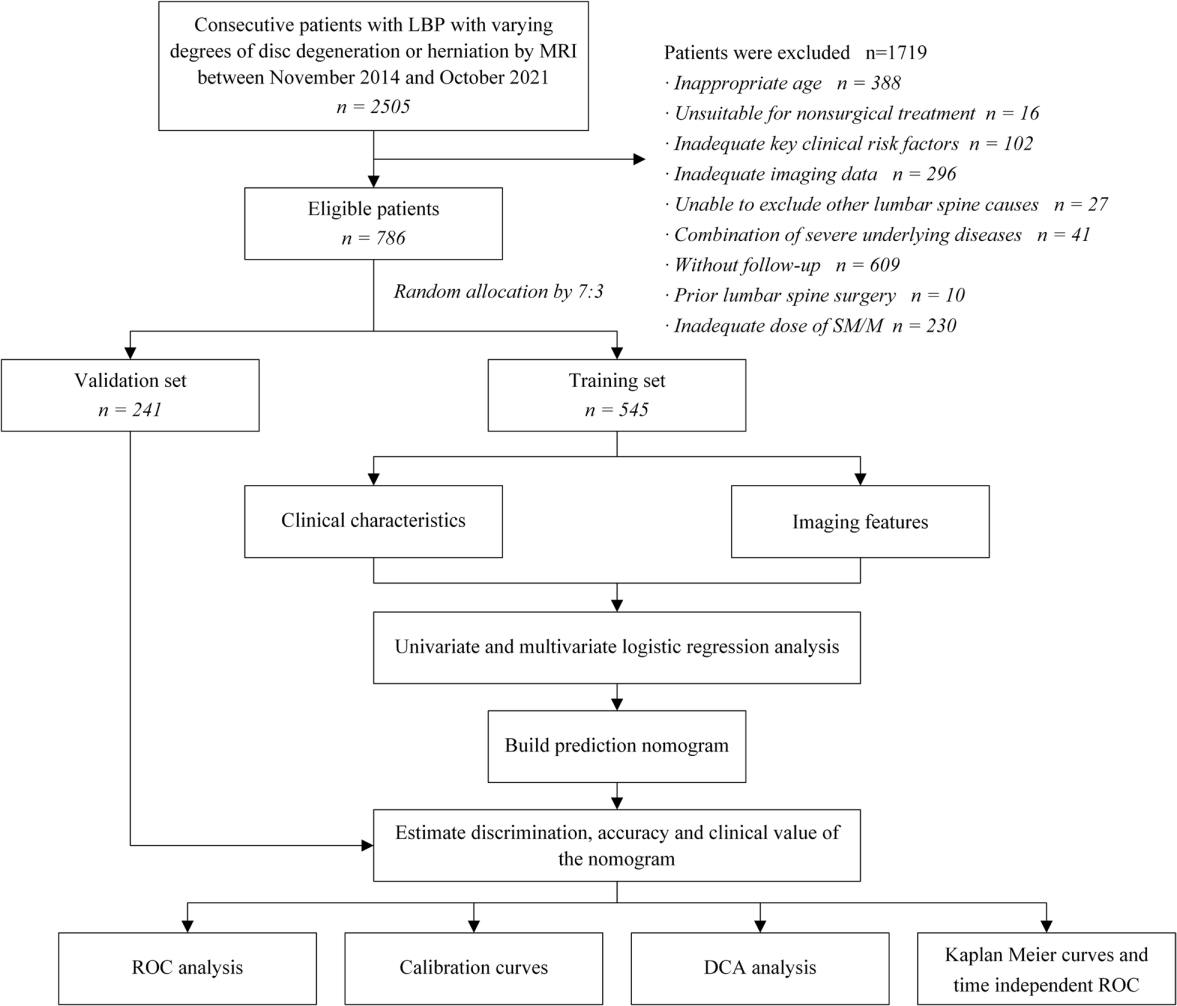
The patient selection process and analysis flowchart of this study. LBP, low back pain; MRI, magnetic resonance imaging; SM/M, spinal manipulation/mobilization; ROC, receiver operating characteristic; DCA, Decision curve analysis

In total, 786 consecutive patients with LBP who met the inclusion criteria and were treated in our hospital between November 2014 and October 2021 were included. MRI confirmed varying degrees of disc degeneration or herniation, with or without radicular pain. Subsequently, 545 and 241 patients were assigned to the training and validation sets, respectively (Fig. 1).

Among the 786 patients included in the retrospective cohort for model development, the mean age was 49.05 years (standard deviation (SD) 12.38 years), with 45.93% males and 54.07% females. The highest number of herniated disc segments was observed in lumbar (L) 5-sacral (S) 1 (49.36%), followed by L4–L5 (44.78%), and other (5.85%). The Pfirrmann classification of disc degeneration found that grades III (31.81%) and VI (32.95%) were the most common, followed by grades II (12.09%), V (17.43%), with VI (4.20%) and VII (1.52%) being the least common. There was no important difference in clinical characteristics and imaging features between the two sets, supporting their use as training and validation sets (Additional file 4: Appendix S4).

### Independent prognostic factor screening and nomogram construction

**Fig. 2:**
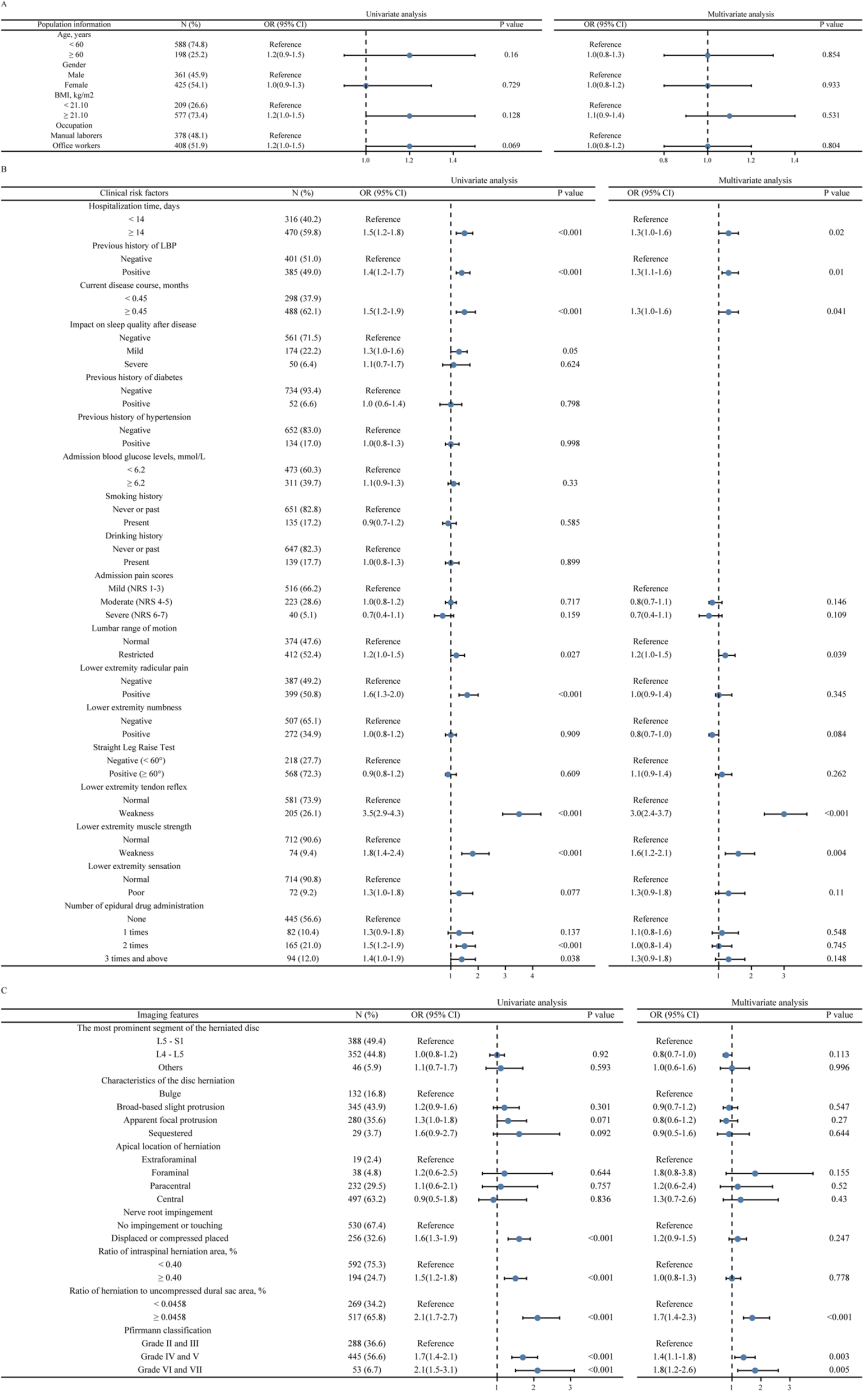
Forest plots for univariate and multivariable analysis of recurrence in the training set. Based on population information (**A**), clinical risk factors (**B**) and imaging features (**C**). BMI, body mass index; LBP, low back pain; NRS, numeric rating scales

Univariate analysis revealed that clinical characteristics, including hospitalization time (*P* < 0.001), previous history of LBP (*P* < 0.001), disease duration (*P* < 0.001), lumbar range of motion (*P* = 0.027), lower extremity radicular pain (*P* < 0.001), lower extremity tendon reflex and muscle strength (*P* < 0.001), and information collected on MRI—such as nerve root impingement (*P* < 0.001), ratio of intraspinal herniation area (*P* = 0.003), ratio of herniation to uncompressed dural sac area (*P* = 0.018), and Pfirrmann classification (*P* < 0.001)—were associated with the recurrence of LBP after SM/M at 1-year follow-up (Fig. 2A–C, Additional file 5: Appendix S5).

**Fig. 3:**
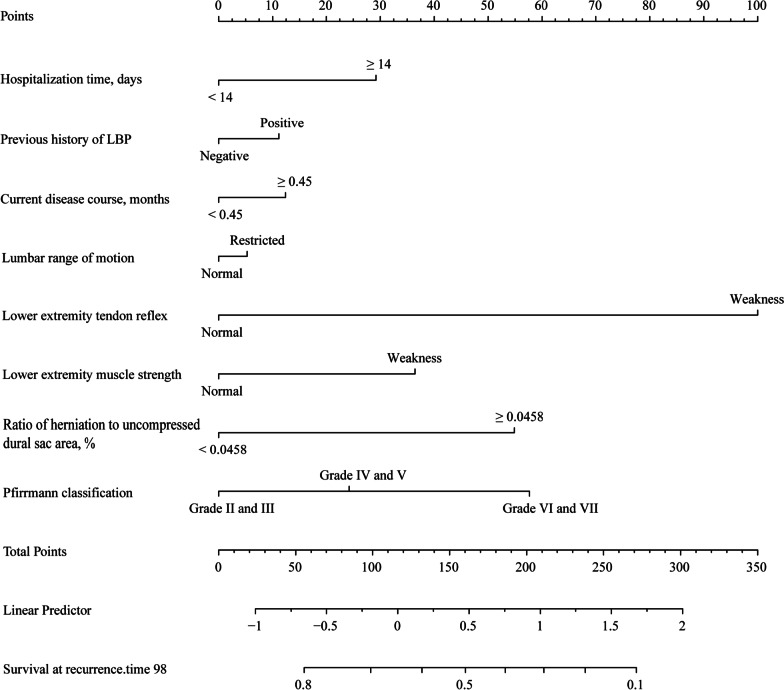
Nomogram for predicting the recurrence of LBP after SM/M at the 1-year follow-up. The Score for each predictor is obtained by drawing a vertical line upward to the points line, and the sum of the scores is calculated by summing the scores associated with these predictors and identified on the total points line. LBP, low back pain

Factors (*P* > 0.05) such as age, sex, BMI, occupation, straight leg raise test, apical location of herniation, and characteristics of the disc herniation were analyzed with the indicators described above in a multivariable analysis because they were considered to have valuable clinical significance. Finally, hospitalization time (*P* = 0.016), previous history of LBP (*P* = 0.016), disease duration (*P* = 0.040), lumbar range of motion (*P* = 0.029), lower extremity tendon reflex (*P* < 0.001), muscle strength (*P* = 0.005), ratio of herniation to uncompressed dural sac area (*P* < 0.001), and Pfirrmann classification (*P* < 0.001) were identified as predictors of recurrence (Fig. 2A–C, Additional file 5: Appendix S5). Next, we constructed a nomogram based on these predictors (Fig. 3).

### Performance and validation of the nomogram

**Fig. 4:**
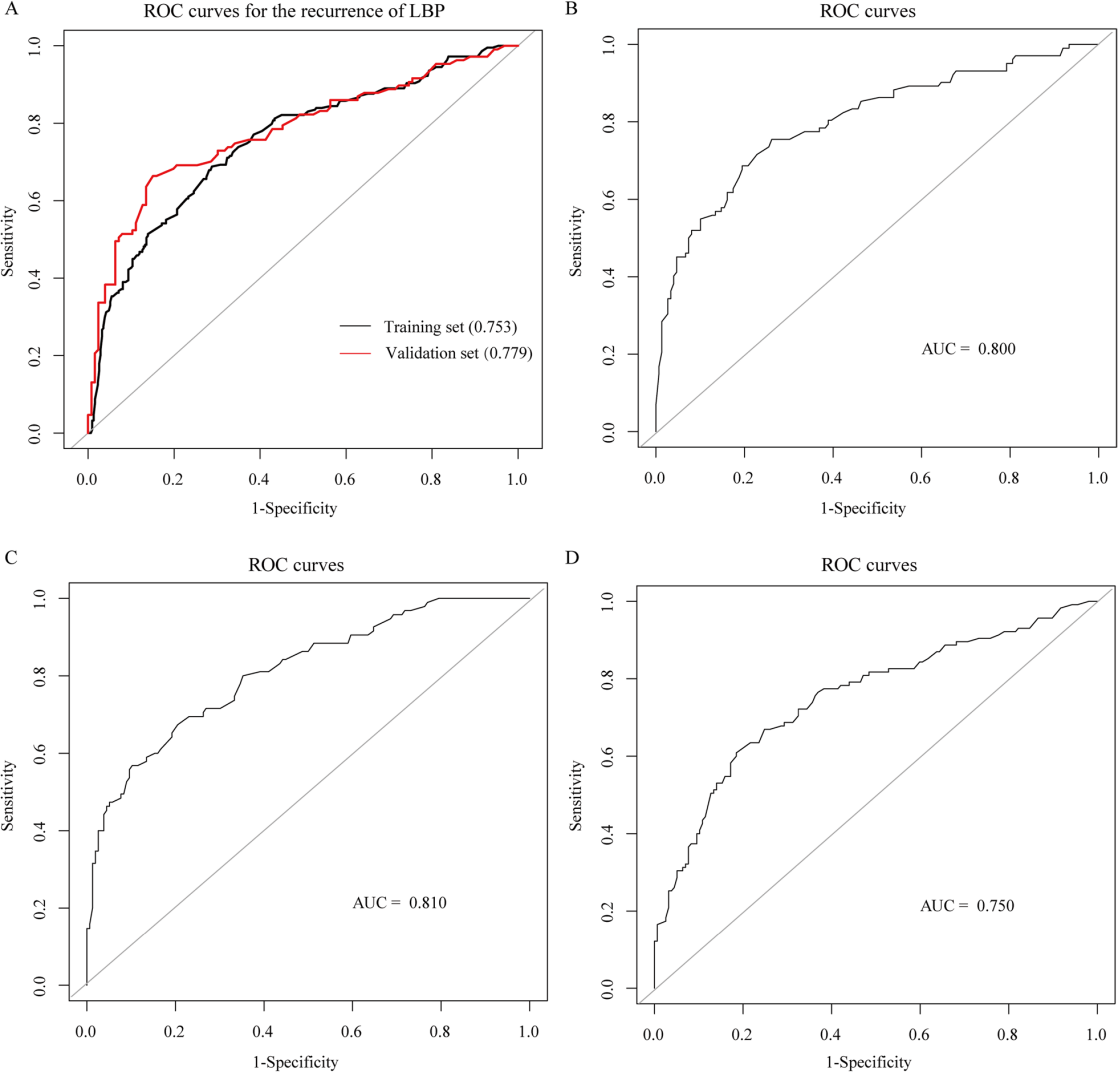
ROC for prediction of the recurrence of LBP after SM/M at the 1-year follow-up. The nomogram used in the training and validation set (**A**). And subgroups of the male (**B**), manual laborers (**C**), and L5-S1 disc herniation (**D**) in the training set. ROC, receiver operating characteristic; LBP, low back pain

**Fig. 5:**
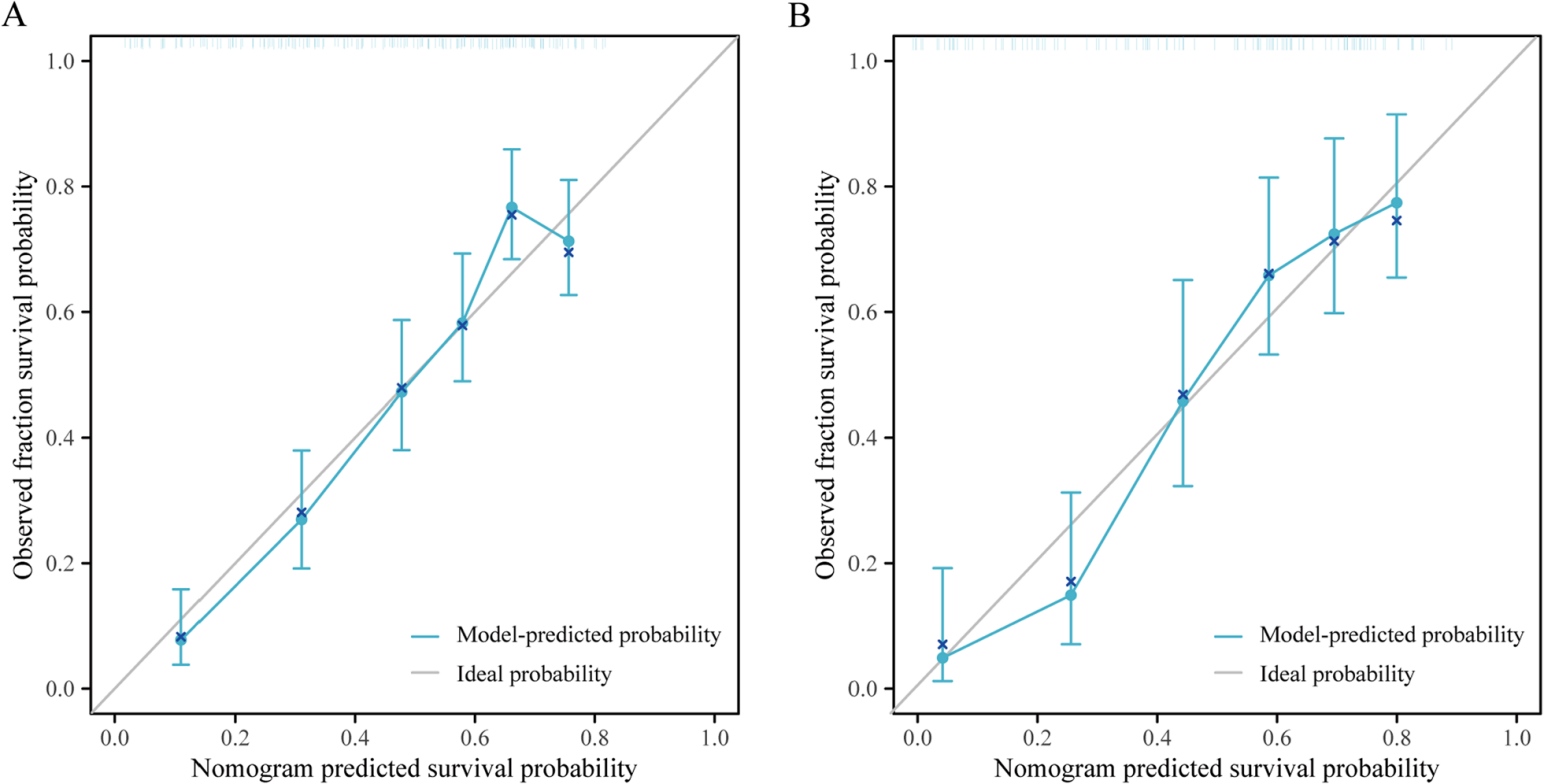
The calibration curves of the nomogram for prediction of the recurrence of LBP after SM/M at the 1-year follow-up. In the training set (**A**) and the validation set (**B**). Vertical axis: the observed probability of recurrence; horizontal axis: the nomogram predicted recurrence probabilities. LBP, low back pain

The C-index for the nomogram predicting LBP recurrence after SM/M at 1-year follow-up was 0.753 (95% CI 0.733–0.806) in the training and 0.779 (95% CI 0.725–0.833) in the validation (Fig. 4A) set. We also performed subgroup analyses to validate the constructed model for males, manual laborers, and the most serious lumbar disc herniation segment—L5-S1. The corresponding C-indices for the prediction of these three models were 0.800, 0.810, and 0.750 in the training set (Fig. 4B–D). Calibration plots of the training and validation sets all graphically showed good agreement between the actual, confirmed by follow-up, and predicted risk of LBP recurrence after SM/M in both the training and validation sets (Fig. 5A, B).

**Fig. 6:**
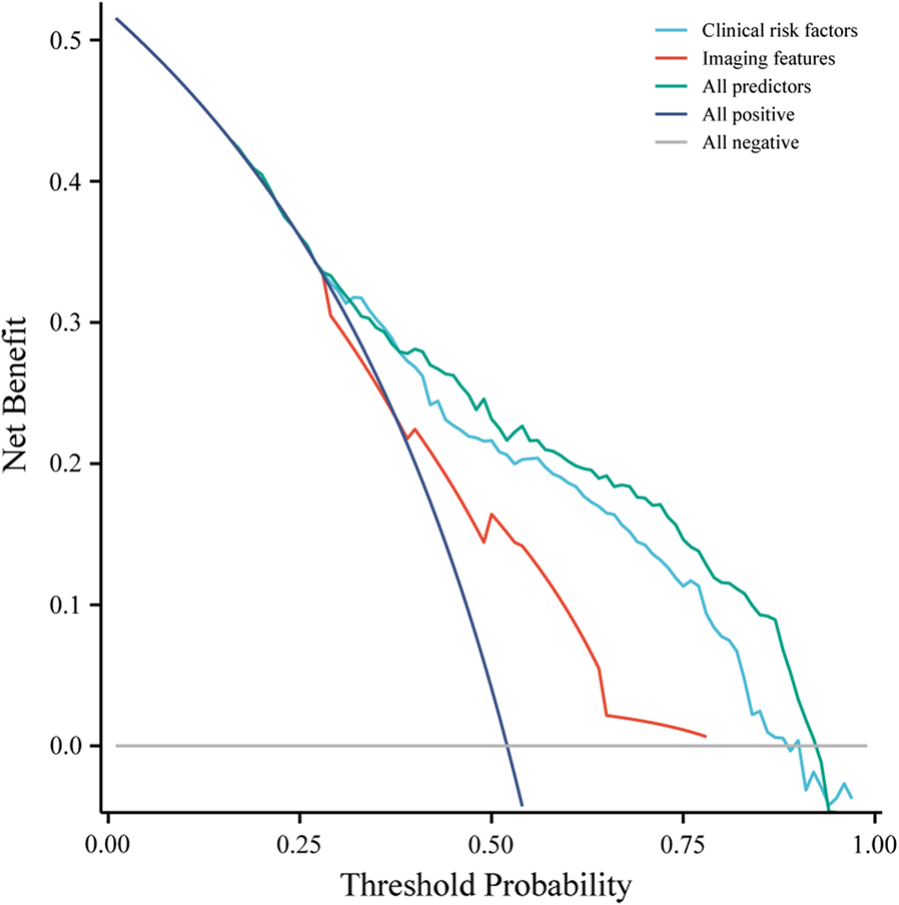
The decision curves of the nomogram for prediction of recurrence of LBP after SM/M at the 1-year follow-up in overall patients. Vertical axis: the net benefit; horizontal axis: the threshold probability at a range of 0.0 to 1.0. The gray line represents the decision curve of the assumption that all patients suffer from recurrence; the black line represents the decision curve of the assumption that no patients suffer from recurrence. LBP, low back pain

**Fig. 7:**
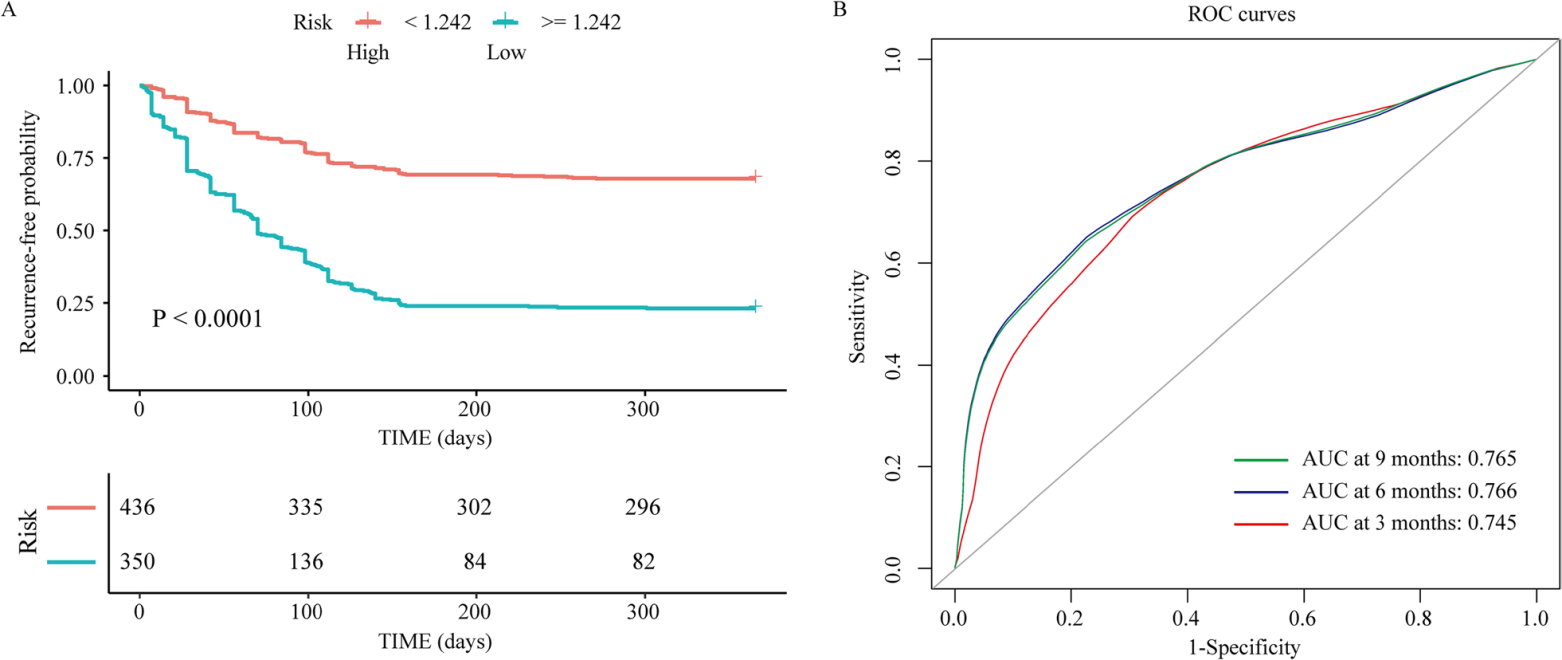
The prognostic value of the nomogram of recurrence-free probability by different risk groups. Recurrence-free probability curves were drawn by the predicted risk status of LBP by the nomogram (**A**). ROC curves for predicted risk status of LBP for predicting recurrence in the total cohort (**B**). LBP, low back pain; ROC, receiver operating characteristic

The DCA of the nomogram for LBP recurrence after SM/M indicated that our nomogram provided more benefits than the treat-all or treat-none schemes (Fig. 6). Figure 7 shows that the predictive efficacy was higher in patients who presented with all identified predictors than those with only clinical factors or imaging features. To evaluate the role of the nomogram we constructed in predicting recurrence, we divided the patients into high- and low-risk groups based on the median recurrence-free probability score (1.242) calculated by the nomogram. Survival analysis showed that patients with a high recurrence-free score (*P* < 0.0001) (Fig. 7A) had a significantly lower recurrence probability than their counterparts. The results of both unadjusted (2.8, 95% CI 2.4–3.2) and adjusted (2.8, 95% CI 2.5–3.3) ROC analyses were consistent with the recurrence-free score. Furthermore, the performance of the predicted risk status at presentation for predicting recurrence at 3-, 6-, and 9-months was 0.745, 0.766, and 0.765, respectively (Fig. 7B).

## Discussion

We established and validated a novel nomogram based on a combination of clinical characteristics and lumbar MRI features to predict the risk of recurrence within 1 year in patients with LBP who have been treated with SM/M. The primary retrospective cohort in this study was obtained from 7 years of inpatient data from the massage department of our hospital. This department represents standard technology or medical services in the field of SM/M in Hangzhou City. Through univariate analysis and subsequent multivariable analysis, we identified hospitalization time, previous history of LBP, disease duration, lumbar range of motion, lower extremity tendon reflex and muscle strength, ratio of herniation to uncompressed dural sac area, and Pfirrmann classification as independent prognostic factors for LBP recurrence after SM/M.

These findings were highly concordant with those of previous reports on risk factors for LBP. A previous history of LBP [29], disease duration [30], and range of motion [31] appear to be associated with symptom severity. Lower extremity tendon reflexes and muscle strength imply the possibility of lumbar radiculopathy [32, 33]. There is also a positive correlation between the ratio of herniation to uncompressed dural sac area and the possibility of mechanical impingement of the nerve root [21]. Notably, the Pfirrmann classification for disc degeneration is an important factor that has been established in many lumbar disorders, and similar studies have supported the relationship between more serious degeneration and worse recurrence-free outcomes [34, 35]. While hospitalization time cannot be used as a prognostic predictor prior to SM/M, it can be used for recurrence prediction. We postulate that the longer the hospital stay, the more adequate the treatment a patient is likely to receive, and the better the clinical outcomes relative to the same discharge criteria.

A meta-analysis of 47 randomized controlled trials (RCTs) [9] and another meta-analysis of 21 RCTs [17] revealed that SM/M produces similar effects as the therapies recommended in the current chronic LBP guidelines. A double-blind RCT demonstrated that active SM/M was more effective than simulated manipulations for pain relief in both acute LBP and sciatica with disc protrusion [36]. However, the above literature and some guidelines remind us that there are some discrepancies in the circumstances under which SM/M should be administered [37, 38]. The main controversy is whether the use of SM/M as a primary treatment option for LBP requires a distinction between patients with acute or chronic pain, with or without radicular pain, and whether it should be administered either alone or preferably in combination with other approaches [39]. These clinical obstacles have created the need for new and improved therapeutic strategies.

Unlike other clinical trials investigating SM/M for LBP, our inclusion criteria [36, 40] allowed patients to be precisely selected from clinical practice. Each case had a detailed history and neurological examination to ensure that they did not require emergency surgery (to ensure the safety of the SM/M). Additionally, patients were recommended for hospitalization and spinal MRI because of their very high pain levels or the possibility of invasive procedures with epidural drug administration (to avoid delaying treatment). Further, the varying degrees of lumbar disc herniation or degeneration needed to be confirmed based on imaging evidence. Although the etiology of LBP is multifactorial, most causes of LBP can be attributed to the intervertebral disc [41, 42] and include the associated effects of lumbar facet degeneration [43] and myofascial pain [44].

The mechanism of SM/M remains to be determined, but it is likely to be complex and controversial. For the potentially malignant factors mentioned above, the rationale for manipulation is recognized to be the correction of disc displacement, release of adhesive fibrosis surrounding prolapsed discs or facet joints, entrapped synovial folds or plicae, relaxation of hypertonic muscles, and unbuckling displaced motion segments [36, 45]. SM/M practice may also be interpreted in various ways in different countries, but the basic principle is to restore and protect the disturbed neuromusculoskeletal system of patients with LBP [9].

Therefore, we sought to develop a nomogram to predict recurrence-free conditions in non-surgical LBP patients after SM/M and identify participants who would respond better to SM/M. Our prognostic model indicated that patients with LBP with a first episode, short-term disease course, no limitation of movement or lumbar radiculopathy, minor disc herniation, or mild degeneration would be better treated with SM/M. It revealed good calibration and discriminative power after internal validation; however, external validation is recommended before implementation in clinical practice. Unlike previous studies investigating the recurrence of LBP after SM/M, our most significant improvement was the validation of predictors [46]. Knecht et al. [47] and Petrozzi et al. [48] identified the potential predictive value of disease duration and work ability in the recovery of LBP after SM/M. Our nomogram was further refined by adding not only clinically relevant predictors but also introducing lumbar imaging features. This may facilitate a more individualized treatment approach.

## Study limitations

This study has some limitations. First, it is retrospective and included participants from a single institution. Thus, prospective cohort-based analyses and external validation of additional sites are required. Second, we did not draw precise etiological subgroup distinctions for LBP, nor is there a good distinction between acute and chronic LBP. This is another reason prospective investigations are required to further confirm the reliability of our nomogram. To better understand the effectiveness of SM/M, a more detailed prognostication among different LBP types needs to be performed. Finally, research incorporating radiomics into predictive nomograms should be conducted in the future, as both qualitative and quantitative imaging features suffer from certain constraints.

## Conclusion

A nomogram incorporating clinical factors and imaging features achieved satisfactory performance in individualized prediction of the 1-year recurrence-free period of LBP after SM/M, allowing optimization of the non-surgical treatment strategy in patients. Further validation is required in future prospective studies and with data from external cohorts.

### Supplementary Information

**Additional file 1:** TRIPOD Checklist: Prediction Model Development and Validation.

**Additional file 2:** Spinal manipulation/mobilization.

**Additional file 3:** Data acquisition method of imaging features.

**Additional file 4:** Comparisons of clinical characteristics and imaging features of patients in the training and validation set.

**Additional file 5:** Univariate and multivariate analysis of recurrence based on population information, clinical risk factors and imaging features in the training set.

## Data Availability

The dataset used within this study is available from the corresponding author on a reasonable request.

## Abbreviations

AUC: Area under the curve
BMI: Body mass index
DCA: Decision curve analysis
LBP: Low back pain
MRI: Magnetic resonance imaging
RCT: Randomized controlled trial
ROC: Receiver operating characteristic
SM/M: Spinal manipulation/mobilization
TCM: Traditional Chinese medicine
