# Supplementary material for: A multidimensional nomogram combining clinical factors and imaging features to predict 1-year recurrence of low back pain with or without radicular pain after spinal manipulation/mobilization
Source: Chiropr Man Therap. 2023 Aug 10;31:27. doi: 10.1186/s12998-023-00500-5 (PMC10416529; doi:10.1186/s12998-023-00500-5)
Supplement: Supplementary file 1 — Additional file 1: TRIPOD Checklist: Prediction Model Development and Validation. [file 12998_2023_500_MOESM1_ESM.docx]

| **Section/Topic** | **Item** |  | **Checklist Item** | **Page** |
| --- | --- | --- | --- | --- |
| **Title and abstract** | | | | |
| Title | 1 | D;V | Identify the study as developing and/or validating a multivariable prediction model, the target population, and the outcome to be predicted. | Page1/Line1-3 |
| Abstract | 2 | D;V | Provide a summary of objectives, study design, setting, participants, sample size, predictors, outcome, statistical analysis, results, and conclusions. | Page2-3/Line14-43 |
| **Introduction** | | | | |
| Background and objectives | 3a | D;V | Explain the medical context (including whether diagnostic or prognostic) and rationale for developing or validating the multivariable prediction model, including references to existing models. | Page4-5/Line48-86 |
|  | 3b | D;V | Specify the objectives, including whether the study describes the development or validation of the model or both. | Page5/Line 87-89 |
| **Methods** | | | | |
| Source of data | 4a | D;V | Describe the study design or source of data (e.g., randomized trial, cohort, or registry data), separately for the development and validation data sets, if applicable. | Page5-6/Line96-98 |
|  | 4b | D;V | Specify the key study dates, including start of accrual; end of accrual; and, if applicable, end of follow-up. | Page6/Line100-101 |
| Participants | 5a | D;V | Specify key elements of the study setting (e.g., primary care, secondary care, general population) including number and location of centres. | Page6/Line98-103 |
|  | 5b | D;V | Describe eligibility criteria for participants. | Page6/Line104-113 |
|  | 5c | D;V | Give details of treatments received, if relevant. | Page6/Line107-108 |
| Outcome | 6a | D;V | Clearly define the outcome that is predicted by the prediction model, including how and when assessed. | Page6-7/Line114-138 |
|  | 6b | D;V | Report any actions to blind assessment of the outcome to be predicted. | Page7/Line126-138 |
| Predictors | 7a | D;V | Clearly define all predictors used in developing or validating the multivariable prediction model, including how and when they were measured. | Page6-7/Line114-138 |
|  | 7b | D;V | Report any actions to blind assessment of predictors for the outcome and other predictors. | Page7/Line126-138 |
| Sample size | 8 | D;V | Explain how the study size was arrived at. | N/A. All patients were retrieved from the medical information system of our hospital from November 2014 to October 2021. Low back pain patients who met the inclusion standard have all been included. Therefore, the patients' number was determined. |
| Missing data | 9 | D;V | Describe how missing data were handled (e.g., complete-case analysis, single imputation, multiple imputation) with details of any imputation method. | Page6/Line109 |
| Statistical analysis methods | 10a | D | Describe how predictors were handled in the analyses. | Page7-8/Line140-147 |
|  | 10b | D | Specify type of model, all model-building procedures (including any predictor selection), and method for internal validation. | Page8/Line148-154 |
|  | 10c | V | For validation, describe how the predictions were calculated. | Page8/Line156-157 |
|  | 10d | D;V | Specify all measures used to assess model performance and, if relevant, to compare multiple models. | Page8/Line157-162 |
|  | 10e | V | Describe any model updating (e.g., recalibration) arising from the validation, if done. | Page8-9/Line162-166 |
| Risk groups | 11 | D;V | Provide details on how risk groups were created, if done. | Page9/Line171-184 |
| Development vs. validation | 12 | V | For validation, identify any differences from the development data in setting, eligibility criteria, outcome, and predictors. | Page9/Line167-170 |
| **Results** | | | | |
| Participants | 13a | D;V | Describe the flow of participants through the study, including the number of participants with and without the outcome and, if applicable, a summary of the follow-up time. A diagram may be helpful. | Page9-10/Line188-192 |
|  | 13b | D;V | Describe the characteristics of the participants (basic demographics, clinical features, available predictors), including the number of participants with missing data for predictors and outcome. | Page10/Line193-200 |
|  | 13c | V | For validation, show a comparison with the development data of the distribution of important variables (demographics, predictors and outcome). | Page10/Line200-203 |
| Model development | 14a | D | Specify the number of participants and outcome events in each analysis. | Page10-11/Line205-224 |
|  | 14b | D | If done, report the unadjusted association between each candidate predictor and outcome. | N/A |
| Model specification | 15a | D | Present the full prediction model to allow predictions for individuals (i.e., all regression coefficients, and model intercept or baseline survival at a given time point). | Page11/Line226-228 |
|  | 15b | D | Explain how to the use the prediction model. | Page11-12/Line228-241 |
| Model performance | 16 | D;V | Report performance measures (with CIs) for the prediction model. | Page12/Line242-247 |
| Model-updating | 17 | V | If done, report the results from any model updating (i.e., model specification, model performance). | N/A |
| **Discussion** | | | | |
| Limitations | 18 | D;V | Discuss any limitations of the study (such as nonrepresentative sample, few events per predictor, missing data). | Page15-16/Line318-327 |
| Interpretation | 19a | V | For validation, discuss the results with reference to performance in the development data, and any other validation data. | Page12-13/Line250-272 |
|  | 19b | D;V | Give an overall interpretation of the results, considering objectives, limitations, results from similar studies, and other relevant evidence. | Page13-15/Line273-303 |
| Implications | 20 | D;V | Discuss the potential clinical use of the model and implications for future research. | Page15/Line304-316 |
| **Other information** | | | | |
| Supplementary information | 21 | D;V | Provide information about the availability of supplementary resources, such as study protocol, Web calculator, and data sets. | Supplementary appendices 1-5 |
| Funding | 22 | D;V | Give the source of funding and the role of the funders for the present study. | Page17/Line350-352 |

*Items relevant only to the development of a prediction model are denoted by D, items relating solely to a validation of a prediction model are denoted by V, and items relating to both are denoted D;V. We recommend using the TRIPOD Checklist in conjunction with the TRIPOD Explanation and Elaboration document.
