# Supplementary material for: A multidimensional nomogram combining clinical factors and imaging features to predict 1-year recurrence of low back pain with or without radicular pain after spinal manipulation/mobilization
Source: Chiropr Man Therap. 2023 Aug 10;31:27. doi: 10.1186/s12998-023-00500-5 (PMC10416529; doi:10.1186/s12998-023-00500-5)
Supplement: Supplementary file 2 — Additional file 2: Spinal manipulation/mobilization. [file 12998_2023_500_MOESM2_ESM.docx]

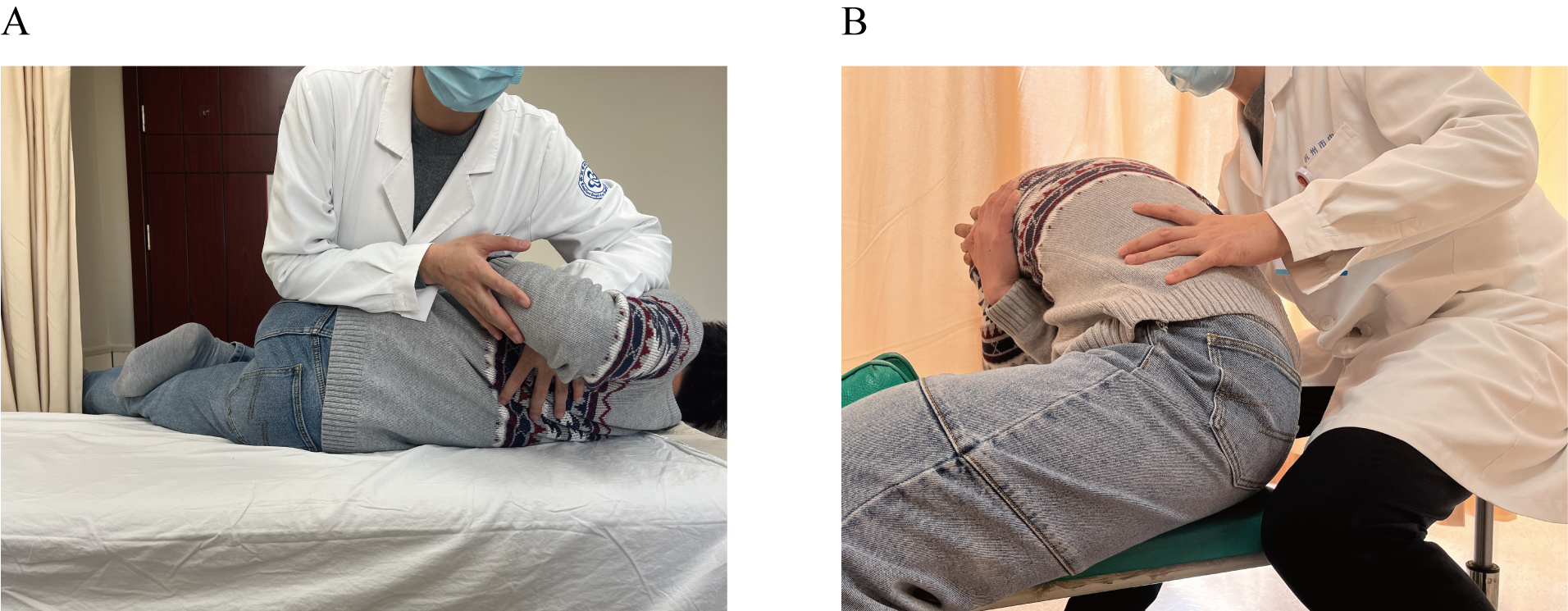


*Supplementary appendix 2.* Spinal manipulation/mobilization. Side reclining (A) and seating position (B).
