# Supplementary material for: A multidimensional nomogram combining clinical factors and imaging features to predict 1-year recurrence of low back pain with or without radicular pain after spinal manipulation/mobilization
Source: Chiropr Man Therap. 2023 Aug 10;31:27. doi: 10.1186/s12998-023-00500-5 (PMC10416529; doi:10.1186/s12998-023-00500-5)
Supplement: Supplementary file 3 — Additional file 3: Data acquisition method of imaging features. [file 12998_2023_500_MOESM3_ESM.docx]

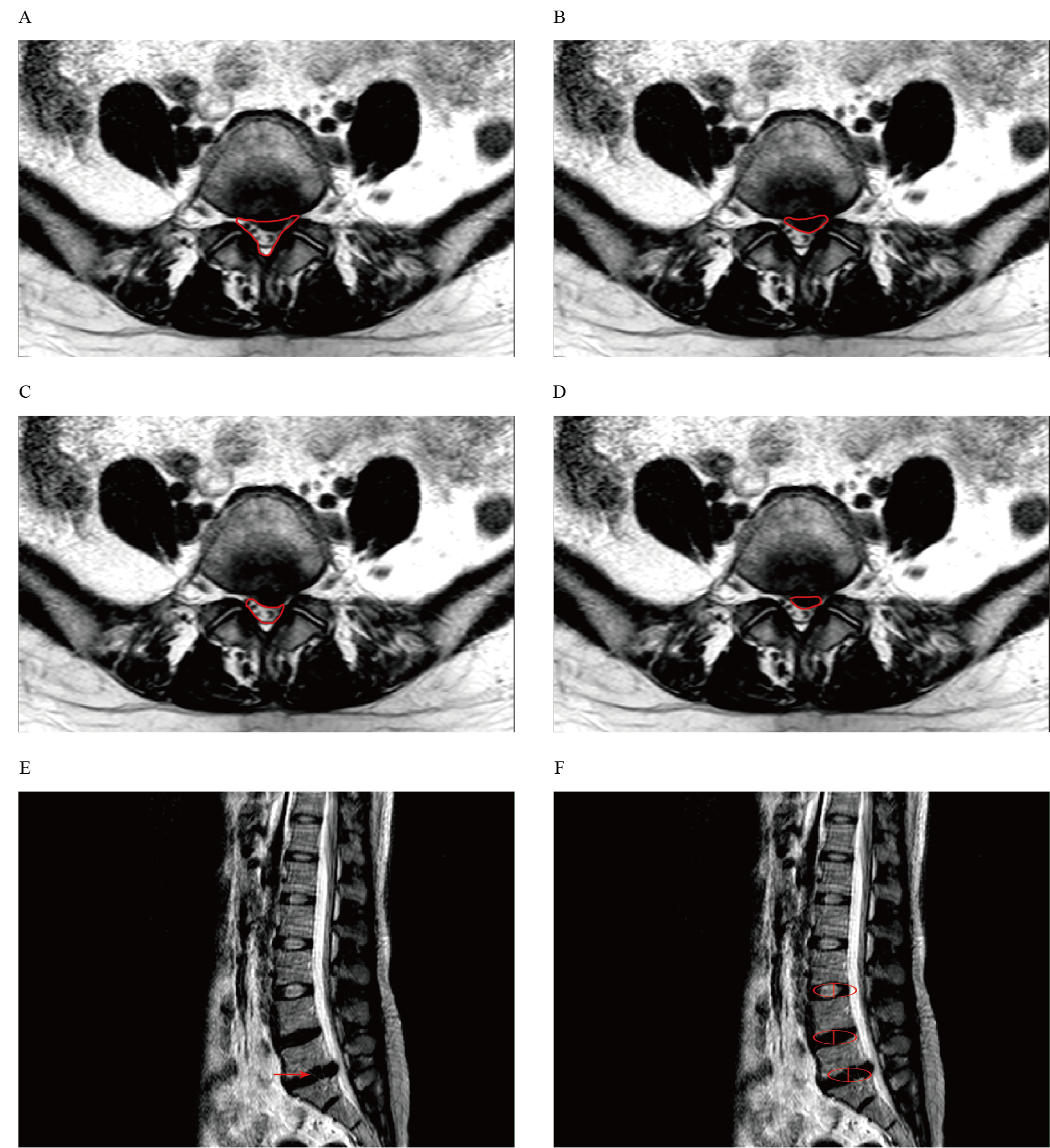


*Supplementary appendix 3.* Data acquisition method of imaging features. The ratio of intraspinal herniation area was derived from (B) divided by the area of the red circle of (A). The ratio of herniation to uncompressed dural sac area was derived from (D) divided by the area of the red circle of (C). T2WI signal intensity of the epidural material (E) and maximum height of intervertebral disc (F) in sagittal.
