# Supplementary material for: A multidimensional nomogram combining clinical factors and imaging features to predict 1-year recurrence of low back pain with or without radicular pain after spinal manipulation/mobilization
Source: Chiropr Man Therap. 2023 Aug 10;31:27. doi: 10.1186/s12998-023-00500-5 (PMC10416529; doi:10.1186/s12998-023-00500-5)
Supplement: Supplementary file 4 — Additional file 4: Comparisons of clinical characteristics and imaging features of patients in the training and validation set. [file 12998_2023_500_MOESM4_ESM.docx]

*Supplementary appendix 4.* Comparisons of clinical characteristics and imaging features of patients in the training and validation set.

| Variable | Training set  (N=545) (%) | Validation set  (N=241) (%) |
| --- | --- | --- |
| Age, years |  |  |
| < 60 | 414 (76.0) | 174 (72.2) |
| ≥ 60 | 131 (24.0) | 67 (27.8) |
| Gender |  |  |
| Male | 254 (46.6) | 107 (44.4) |
| Female | 291 (53.4) | 134 (55.6) |
| BMI, kg/m^2^ |  |  |
| < 21.10 | 152 (27.9) | 57 (23.7) |
| ≥ 21.10 | 393 (72.1) | 184 (76.3) |
| Occupation |  |  |
| Manual laborers | 255 (46.8) | 123 (51.0) |
| Office workers | 290 (53.2) | 118 (49.0) |
| Hospitalization time, days |  |  |
| < 14 | 229 (42.0) | 87 (36.1) |
| ≥ 14 | 316 (58.0) | 154 (63.9) |
| Previous history of LBP |  |  |
| Negative | 286 (52.5) | 115 (47.7) |
| Positive | 259 (47.5) | 126 (52.3) |
| Disease duration, months |  |  |
| < 0.45 | 204 (37.4) | 94 (39.0) |
| ≥ 0.45 | 341 (62.6) | 147 (61.0) |
| Impact on sleep quality after disease |  |  |
| Negative | 396 (72.7) | 165 (68.8) |
| Mild | 118 (21.7) | 56 (23.3) |
| Severe | 31 (5.7) | 19 (7.9) |
| Previous history of diabetes |  |  |
| Negative | 514 (94.3) | 220 (91.3) |
| Positive | 31 (5.7) | 21 (8.7) |
| Previous history of hypertension |  |  |
| Negative | 448 (82.2) | 204 (84.6) |
| Positive | 97 (17.8) | 37 (15.4) |
| Previous history of cardiopathy |  |  |
| Negative | 539 (98.9) | 237 (98.3) |
| Positive | 6 (1.1) | 4 (1.7) |
| Admission blood glucose levels, mmol/L |  |  |
| < 6.2 | 326 (59.8) | 147 (61.5) |
| ≥ 6.2 | 219 (40.2) | 92 (38.5) |
| Smoking history |  |  |
| Never or past | 443 (81.3) | 208 (86.3) |
| Present | 102 (18.7) | 33 (13.7) |
| Drinking history |  |  |
| Never or past | 439 (80.6) | 208 (86.3) |
| Present | 106 (19.4) | 33 (13.7) |
| Admission pain scores |  |  |
| Mild (NRS 1-3) | 363 (67.5) | 153 (63.5) |
| Moderate (NRS 4-5) | 152 (28.3) | 71 (29.5) |
| Severe (NRS 6-7) | 23 (4.3) | 17 (7.1) |
| Lumbar range of motion |  |  |
| Normal | 253 (46.4) | 121 (50.2) |
| Restricted | 292 (53.6) | 120 (49.8) |
| Lower extremity radicular pain |  |  |
| Negative | 263 (48.3) | 124 (51.5) |
| Positive | 282 (51.7) | 117 (48.5) |
| Lower extremity numbness |  |  |
| Negative | 349 (64.9) | 158 (65.6) |
| Positive | 189 (35.1) | 83 (34.4) |
| Straight Leg Raise Test |  |  |
| Negative (< 60°) | 158 (29.0) | 60 (24.9) |
| Positive (≥ 60°) | 387 (71.0) | 181 (75.1) |
| Lower extremity tendon reflex |  |  |
| Normal | 403 (73.9) | 178 (73.9) |
| Weakness | 142 (26.1) | 63 (26.1) |
| Lower extremity muscle strength |  |  |
| Normal | 496 (91.0) | 216 (89.6) |
| Weakness | 49 (9.0) | 25 (10.4) |
| Lower extremity sensation |  |  |
| Normal | 498 (91.4) | 216 (89.6) |
| Poor | 47 (8.6) | 25 (10.4) |
| Number of epidural administration |  |  |
| None | 310 (56.9) | 135 (56.0) |
| 1 times | 55 (10.1) | 27 (11.2) |
| 2 times | 116 (21.3) | 49 (20.3) |
| 3 times and above | 64 (11.7) | 30 (12.5) |
| The most prominent segment of the herniated disc |  |  |
| L5 - S1 | 262 (48.1) | 126 (52.3) |
| L4 - L5 | 253 (46.4) | 99 (41.1) |
| Others | 30 (5.5) | 16 (6.6) |
| Characteristics of the disc herniation |  |  |
| Bulge | 92 (16.9) | 40 (16.6) |
| Broad-based slight protrusion | 242 (44.4) | 103 (42.7) |
| Apparent focal protrusion | 189 (34.7) | 91 (37.8) |
| Sequestered | 22 (4.0) | 7 (2.9) |
| Apical location of herniation |  |  |
| Extraforaminal | 16 (2.9%) | 3 (1.2%) |
| Foraminal | 26 (4.8%) | 12 (5.0%) |
| Paracentral | 157 (28.8%) | 75 (31.1%) |
| Central | 346 (63.5%) | 151 (62.7%) |
| Nerve root impingement |  |  |
| No impingement or touching | 376 (69.0) | 154 (63.9) |
| Displaced or compressed placed | 169 (31.0) | 87 (36.1) |
| Ratio of intraspinal herniation area, % |  |  |
| < 0.40 | 408 (74.9) | 184 (76.3) |
| ≥ 0.40 | 137 (25.1) | 57 (23.7) |
| Ratio of herniation to uncompressed dural sac area, % |  |  |
| < 0.0458 | 179 (32.8) | 90 (37.3) |
| ≥ 0.0458 | 366 (67.2) | 151 (62.7) |
| Pfirrmann classification |  |  |
| Grade II and III | 204 (37.4) | 84 (34.9) |
| Grade IV and V | 305 (56.0) | 140 (58.1) |
| Grade VI and VII | 36 (6.6) | 17 (7.1) |
| 1-year follow-up |  |  |
| Normal | 263 (48.3) | 114 (47.3) |
| Recurrence | 282 (51.7) | 127 (52.7) |

BMI, body mass index; LBP, low back pain; NRS, numeric rating scales.
