# Supplementary material for: A multidimensional nomogram combining clinical factors and imaging features to predict 1-year recurrence of low back pain with or without radicular pain after spinal manipulation/mobilization
Source: Chiropr Man Therap. 2023 Aug 10;31:27. doi: 10.1186/s12998-023-00500-5 (PMC10416529; doi:10.1186/s12998-023-00500-5)
Supplement: Supplementary file 5 — Additional file 5: Univariate and multivariate analysis of recurrence based on population information, clinical risk factors and imaging features in the training set. [file 12998_2023_500_MOESM5_ESM.docx]

*Supplementary appendix 5.* Univariate and multivariate analysis of recurrence based on population information, clinical risk factors and imaging features in the training set.

| Risk factor | Univariate analysis | | |  | Multivariate analysis | | |
| --- | --- | --- | --- | --- | --- | --- | --- |
|  | OR | 95% | P value |  | OR | 95% | P value |
| Population information | | | | | | | |
| Age, years |  |  |  |  |  |  |  |
| < 60 | 1.0 |  |  |  | 1.0 |  |  |
| ≥ 60 | 1.2 | 0.9-1.5 | 0.160 |  | 1.0 | 0.8-1.3 | 0.854 |
| Gender |  |  |  |  |  |  |  |
| Male | 1.0 |  |  |  | 1.0 |  |  |
| Female | 1.0 | 0.9-1.3 | 0.729 |  | 1.0 | 0.8-1.2 | 0.933 |
| BMI, kg/m^2^ |  |  |  |  |  |  |  |
| < 21.10 | 1.0 |  |  |  | 1.0 |  |  |
| ≥ 21.10 | 1.2 | 1.0-1.5 | 0.128 |  | 1.1 | 0.9-1.4 | 0.531 |
| Occupation |  |  |  |  |  |  |  |
| Manual laborers | 1.0 |  |  |  | 1.0 |  |  |
| Office workers | 1.2 | 1.0-1.5 | 0.069 |  | 1.0 | 0.8-1.2 | 0.804 |
| Clinical risk factors | | | | | | | |
| Hospitalization time, days |  |  |  |  |  |  |  |
| < 14 | 1.0 |  |  |  | 1.0 |  |  |
| ≥ 14 | 1.5 | 1.2-1.8 | <0.001 |  | 1.3 | 1.0-1.6 | 0.020 |
| Previous history of LBP |  |  |  |  |  |  |  |
| Negative | 1.0 |  |  |  | 1.0 |  |  |
| Positive | 1.4 | 1.2-1.7 | <0.001 |  | 1.3 | 1.1-1.6 | 0.010 |
| Disease duration, months |  |  |  |  |  |  |  |
| < 0.45 | 1.0 |  |  |  | 1.0 |  |  |
| ≥ 0.45 | 1.5 | 1.2-1.9 | <0.001 |  | 1.3 | 1.0-1.6 | 0.041 |
| Impact on sleep quality after disease |  |  |  |  |  |  |  |
| Negative | 1.0 |  |  |  |  |  |  |
| Mild | 1.3 | 1.0-1.6 | 0.050 |  |  |  |  |
| Severe | 1.1 | 0.7-1.7 | 0.624 |  |  |  |  |
| Previous history of diabetes |  |  |  |  |  |  |  |
| Negative | 1.0 |  |  |  |  |  |  |
| Positive | 1.0 | 0.6-1.4 | 0.798 |  |  |  |  |
| Previous history of hypertension |  |  |  |  |  |  |  |
| Negative | 1.0 |  |  |  |  |  |  |
| Positive | 1.0 | 0.8-1.3 | 0.998 |  |  |  |  |
| Previous history of cardiopathy |  |  |  |  |  |  |  |
| Negative | 1.0 |  |  |  |  |  |  |
| Positive | 1.4 | 0.6-3.2 | 0.381 |  |  |  |  |
| Admission blood glucose levels, mmol/L |  |  |  |  |  |  |  |
| < 6.2 | 1.0 |  |  |  |  |  |  |
| ≥ 6.2 | 1.1 | 0.9-1.3 | 0.330 |  |  |  |  |
| Smoking history |  |  |  |  |  |  |  |
| Never or past | 1.0 |  |  |  |  |  |  |
| Present | 0.9 | 0.7-1.2 | 0.585 |  |  |  |  |
| Drinking history |  |  |  |  |  |  |  |
| Never or past | 1.0 |  |  |  |  |  |  |
| Present | 1.0 | 0.8-1.3 | 0.899 |  |  |  |  |
| Admission pain scores |  |  |  |  |  |  |  |
| Mild (NRS 1-3) | 1.0 |  |  |  | 1.0 |  |  |
| Moderate (NRS 4-5) | 1.0 | 0.8-1.2 | 0.717 |  | 0.8 | 0.7-1.1 | 0.146 |
| Severe (NRS 6-7) | 0.7 | 0.4-1.1 | 0.159 |  | 0.7 | 0.4-1.1 | 0.109 |
| Lumbar range of motion |  |  |  |  |  |  |  |
| Normal | 1.0 |  |  |  | 1.0 |  |  |
| Restricted | 1.2 | 1.0-1.5 | 0.027 |  | 1.2 | 1.0-1.5 | 0.039 |
| Lower extremity radicular pain |  |  |  |  |  |  |  |
| Negative | 1.0 |  |  |  | 1.0 |  |  |
| Positive | 1.6 | 1.3-2.0 | <0.001 |  | 1.0 | 0.9-1.4 | 0.345 |
| Lower extremity numbness |  |  |  |  |  |  |  |
| Negative | 1.0 |  |  |  | 1.0 |  |  |
| Positive | 1.0 | 0.8-1.2 | 0.909 |  | 0.8 | 0.7-1.0 | 0.084 |
| Straight Leg Raise Test |  |  |  |  |  |  |  |
| Negative (< 60°) | 1.0 |  |  |  | 1.0 |  |  |
| Positive (≥ 60°) | 0.9 | 0.8-1.2 | 0.609 |  | 1.1 | 0.9-1.4 | 0.262 |
| Lower extremity tendon reflex |  |  |  |  |  |  |  |
| Normal | 1.0 |  |  |  | 1.0 |  |  |
| Weakness | 3.5 | 2.9-4.3 | <0.001 |  | 3.0 | 2.4-3.7 | <0.001 |
| Lower extremity muscle strength |  |  |  |  |  |  |  |
| Normal | 1.0 |  |  |  | 1.0 |  |  |
| Weakness | 1.8 | 1.4-2.4 | <0.001 |  | 1.6 | 1.2-2.1 | 0.004 |
| Lower extremity sensation |  |  |  |  |  |  |  |
| Normal | 1.0 |  |  |  | 1.0 |  |  |
| Poor | 1.3 | 1.0-1.8 | 0.077 |  | 1.3 | 0.9-1.8 | 0.110 |
| Number of epidural drug administration |  |  |  |  |  |  |  |
| None | 1.0 |  |  |  | 1.0 |  |  |
| 1 times | 1.3 | 0.9-1.8 | 0.137 |  | 1.1 | 0.8-1.6 | 0.548 |
| 2 times | 1.5 | 1.2-1.9 | <0.001 |  | 1.0 | 0.8-1.4 | 0.745 |
| 3 times and above | 1.4 | 1.0-1.9 | 0.038 |  | 1.3 | 0.9-1.8 | 0.148 |
| Imaging features | | | | | | | |
| The most prominent segment of the herniated disc |  |  |  |  |  |  |  |
| L5 - S1 | 1.0 |  |  |  | 1.0 |  |  |
| L4 - L5 | 1.0 | 0.8-1.2 | 0.920 |  | 0.8 | 0.7-1.0 | 0.113 |
| Others | 1.1 | 0.7-1.7 | 0.593 |  | 1.0 | 0.6-1.6 | 0.996 |
| Characteristics of the disc herniation |  |  |  |  |  |  |  |
| Bulge | 1.0 |  |  |  | 1.0 |  |  |
| Broad-based slight protrusion | 1.2 | 0.9-1.6 | 0.301 |  | 0.9 | 0.7-1.2 | 0.547 |
| Apparent focal protrusion | 1.3 | 1.0-1.8 | 0.071 |  | 0.8 | 0.6-1.2 | 0.270 |
| Sequestered | 1.6 | 0.9-2.7 | 0.092 |  | 0.9 | 0.5-1.6 | 0.644 |
| Apical location of herniation |  |  |  |  |  |  |  |
| Extraforaminal | 1.0 |  |  |  | 1.0 |  |  |
| Foraminal | 1.2 | 0.6-2.5 | 0.644 |  | 1.8 | 0.8-3.8 | 0.155 |
| Paracentral | 1.1 | 0.6-2.1 | 0.757 |  | 1.2 | 0.6-2.4 | 0.520 |
| Central | 0.9 | 0.5-1.8 | 0.836 |  | 1.3 | 0.7-2.6 | 0.430 |
| Nerve root impingement |  |  |  |  |  |  |  |
| No impingement or touching | 1.0 |  |  |  | 1.0 |  |  |
| Displaced or compressed placed | 1.6 | 1.3-1.9 | <0.001 |  | 1.2 | 0.9-1.5 | 0.247 |
| Ratio of intraspinal herniation area, % |  |  |  |  |  |  |  |
| < 0.40 | 1.0 |  |  |  | 1.0 |  |  |
| ≥ 0.40 | 1.5 | 1.2-1.8 | <0.001 |  | 1.0 | 0.8-1.3 | 0.778 |
| Ratio of herniation to uncompressed dural sac area, % |  |  |  |  |  |  |  |
| < 0.0458 | 1.0 |  |  |  | 1.0 |  |  |
| ≥ 0.0458 | 2.1 | 1.7-2.7 | <0.001 |  | 1.7 | 1.4-2.3 | <0.001 |
| Pfirrmann classification |  |  |  |  |  |  |  |
| Grade II and III | 1.0 |  |  |  | 1.0 |  |  |
| Grade IV and V | 1.7 | 1.4-2.1 | <0.001 |  | 1.4 | 1.1-1.8 | 0.003 |
| Grade VI and VII | 2.1 | 1.5-3.1 | <0.001 |  | 1.8 | 1.2-2.6 | 0.005 |

BMI, body mass index; LBP, low back pain; NRS, numeric rating scales.
